# A novel approach to medical school examinations during the COVID-19 pandemic

**DOI:** 10.1080/10872981.2020.1785680

**Published:** 2020-06-29

**Authors:** Elizabeth Birch, Maisie de Wolf

**Affiliations:** Faculty of Life Sciences & Medicine, King’s College London, London, UK

**Keywords:** Medical students, COVID-19, medical school examinations, online examinations, open-book examination

## Abstract

Online teaching for medical students is not an unusual tool used in medical education. Alongside clinical placements, medical students are familiar with online teaching platforms from various members of the faculty. However, the new and necessary method of examining medical students from their own home during the Covid-19 Pandemic is a novel approach. It is vital that medical students continue to be examined, as this establishes the attainment of the curriculum learning outcomes.

## Discussion

As medical students in our penultimate year of study, our teaching program has been significantly impacted by the global pandemic [1,2]. Additionally, as we are based in London one of the most affected regions of the UK, the move to online learning eased the burden on teaching physicians. The presence of medical students on the ward, not acting in a volunteer capacity but exclusively for the purpose of learning, would have posed a needless risk to patients and clinicians [3]. Furthermore, medical students would have required the use of vital personal protective equipment (PPE), of which there were worryingly low stocks to be prioritised for front line staff [4].

Following the cancellation of clinical placements, medical students were forced to adjust quickly to learning entirely from home. Medical schools were hasty to implement online lectures and teaching opportunities despite the global pandemic. Moreover, clinicians were able to continue teaching using online platforms, such as zoom [1]. There has been a strong sense of appreciation towards these clinicians who are both working during the Covid-19 Pandemic yet still finding time to teach. Learning from our predecessors is a valuable and essential way that medical students learn and progress [5].

However, there was apprehension among medical students, and questions were raised regarding how examinations were to take place [2]. Additionally, there were worries of how their mental health would fare, given the prospect of months of online content and revision, alongside concerns regarding their preparedness for life as a qualified doctor [6].

There were various factors that had to be considered regarding the medical school examinations. In addition to establishing whether the medical students have attained the curriculum learning outcomes, the marks were to be used to calculate the Educational Performance Measure (EPM) and were of important value to those wishing to apply for an Academic Fellowship Programme (AFP). Furthermore, any previously arranged Objective Structured Clinical Examinations (OSCEs) had to be cancelled or postponed due to the social distancing measures. The lack of assessment of clinical skills in addition to lack of clinical exposure has led to rising student anxiety and eagerness to return to placement when it is safe to do so. For now, we can only predict how this will impact the future preparedness of junior doctors.

At King’s College London, our medical examination went ahead as planned, but was completed as an open-book examination (OBE) from home via an online system. This format paralleled that of Imperial College London whose final year medical students had recently sat their final year exams during the Covid-19 Pandemic [7]. With the examination going ahead, there remained significant engagement with the online content, with many medical students tuning in to the daily live lectures. This helped with structuring our days and keeping resolve and dedication to learning [8]. Furthermore, with the move to an OBE, the final grade no longer counting towards our EPM, and with previous grades being scaled up, there was a significant reduction in student anxiety.

Online OBEs are something that students across the world are preparing for during the Covid-19 Pandemic. It is an unusual feeling to sit an important examination from your home. Nonetheless, we felt the exam tested our knowledge and closely simulated the reality of having a patient presenting to you. There was not enough time to process all the information given to you and look up every question. The exam was well designed to assess your ability to assimilate all the information given to you and to reach a conclusion, thereby testing your knowledge and problem solving and not your ability to google.
